# AI-Powered Structural and Co-Expression Analysis of Potato (*Solanum tuberosum*) *StABCG25* Transporters Under Drought: A Combined AlphaFold, WGCNA, and MD Approach

**DOI:** 10.3390/biology14121723

**Published:** 2025-12-01

**Authors:** Barış Kurt, Firat Kurt

**Affiliations:** 1 Department of Chemistry, Faculty of Science and Literature, Muş Alparslan University, 49250 Muş, Türkiye; b.kurt@alparslan.edu.tr; 2 Department of Plant Production and Technologies, Faculty of Applied Sciences, Muş Alparslan University, 49250 Muş, Türkiye

**Keywords:** *StABCG25* transporter, AlphaFold, molecular dynamics simulation, WGCNA (weighted gene co-expression network analysis), ABA transport

## Abstract

Drought stress significantly impacts potato (*Solanum tuberosum*) yield and quality, necessitating the identification of molecular regulators involved in stress response. This study presents a systems-level, integrative in silico strategy to characterize *StABCG25* transporter homologs, key players in abscisic acid (ABA) export in Arabidopsis, to evaluate their potential role in drought adaptation. We performed a genome-wide scan of the potato genome and identified four *StABCG25* isoforms. A comprehensive computational framework was applied, including transcriptomic profiling, Weighted Gene Co-expression Network Analysis (WGCNA), AlphaFold2-based 3D modeling, docking, and long-timescale Molecular Dynamics (MD) simulations. Expression analyses revealed the coordinated upregulation of *StABCG25-2* and *-4* in the drought-tolerant FB clone, contrasted by suppression or instability in sensitive cultivars. WGCNA placed *StABCG25-2* as a hub gene in ABA-enriched stress response modules, while *StABCG25-4* was associated with plastid-related pathways, suggesting functional divergence. Structurally, *StABCG25-2* and -6 exhibited high conformational stability in MD simulations, supported by consistent RMSD/RMSF profiles and MM/PBSA-based binding energy estimates. In contrast, *StABCG25-5B*, despite favorable docking scores, demonstrated poor dynamic stability and unreliable binding affinity. Overall, this study highlights the critical role of transcriptional coordination and structural robustness in the functional specialization of *StABCG25* isoforms under drought stress. Our findings underscore the value of combining WGCNA and molecular dynamics simulations to identify structurally and functionally relevant ABA transporters for future crop improvement strategies.

## Simple Summary

Drought is a major problem for potato plants. Some genes help the plant to survive by moving a stress hormone called ABA to where it is needed. In this study, we focused on a group of these genes in potato. Using computer models, we looked at how these genes behave under drought conditions and how stable their protein structures are. We found that two of the genes are more active in drought-tolerant potatoes, and their protein shapes are more stable. These results help us understand how potatoes respond to drought and can guide future efforts to develop stronger potato varieties.

## 1. Introduction

Drought stress is one of the vital factors limiting agricultural productivity. It causes significant losses in both yield and quality of essential crops around the world. Potato (*Solanum tuberosum*), a globally important food crop, is especially sensitive to water shortage. Plants use several defense mechanisms to cope with drought stress, and the phytohormone abscisic acid (ABA) plays a key role in this process by closing stomata to reduce water loss. Therefore, to survive under drought conditions, both the synthesis and transport of ABA are critical [1]. ABA is generally synthesized in vascular tissues and must be transported to guard cells, where its receptors are located. This movement is tightly controlled and depends on specific transport proteins, such as the ATP-binding cassette (ABC) family. Of this family, ABCG25 is identified as one of the ABA transporters in *Arabidopsis thaliana*. ABCG25 functions as an exporter by pumping ABA out of vascular cells. The movement of ABA from phloem companion cells into the apoplast contributes to the regulation of stomatal function [1,2].

The function of AtABCG25 is controlled at multiple levels. While its expression is strongly induced by ABA itself, it is also subject to negative genetic regulation by microRNAs like upu-miR399a [2,3,4]. It is important to note, however, that the ABA transport network exhibits considerable functional redundancy. Studies have shown that mutants lacking several key transporters, including abcg25, can still maintain normal stomatal function, suggesting a complex and resilient system [5]. Furthermore, emerging evidence points to a multifaceted role for ABCG25 beyond ABA transport, implicating it in nutritional stress, pigmentation, disease resistance, and fruit development [6,7,8]. However, drought tolerance is regulated by multiple hormonal pathways (including ABA, JA, ET, SA, and CK) and we focus specifically on ABA transporters because the ABCG25 family plays the most clearly defined role in early ABA-mediated drought signaling.

Despite extensive research on AtABCG25 in Arabidopsis, the functional homologs in agronomically critical crops like potato remain largely uncharacterized. Identifying these proteins and understanding their affinity for ABA is a crucial step toward developing more drought-resilient potato varieties through targeted genetic improvement. This study, therefore, aims to identify and computationally analyze potential ABCG25 homologs in the potato genome. We hypothesize that specific potato ABCG25-like proteins function as ABA transporters and that their interaction with ABA is fundamental to the plant’s drought stress response. To test this hypothesis, we employed a comprehensive in silico strategy. We first identified candidate homologs in the potato genome and then used homology modeling to construct their three-dimensional structures. Subsequently, molecular docking and molecular dynamics (MD) simulations were performed to predict and refine the binding interactions between these proteins and ABA. Computational approaches like MD simulations, coupled with MM/GBSA energy calculations, are powerful tools for investigating the dynamics of protein-ligand binding, often yielding results that are highly consistent with experimental data where such experiments are not feasible [9,10]. The findings from this research provide crucial insights into the efficiency and specificity of potato ABCG25-like proteins, advancing our understanding of their role in plant stress physiology.

## 2. Materials and Methods

### 2.1. Identification and Characterization of ABCG25 Homologs in Potato

To identify potential ABA transporter proteins in potato, the protein sequences of *Arabidopsis thaliana* ABCG25 (UniProt IDs: Q84TH5, Q84TH5-2) were taken from the UniProt database (https://www.uniprot.org, accessed on 20 April 2025) [11]. Each of these sequences was independently used as a query for a BLASTP search against the *Solanum tuberosum* reference proteome (PGSC v3.4) available in the Phytozome v13 database (https://phytozome-next.jgi.doe.gov, accessed on 20 April 2025) [12] (Supplementary Materials, Tables S1 and S2). The searches were performed using default parameters. The resulting hits from both searches were combined, and duplicates were removed. The combined list was then filtered for hits with an E-value smaller than 1 × 10^−30^, an identity of 40% or higher, and a bit score of 118 or more. Through this process, a final set of 23 candidate proteins was identified for further analysis (Supplementary Materials, Table S3).

These 23 candidates were then analyzed for conserved functional domains using Pfam annotations on Motif Search (https://www.genome.jp/tools/motif/, accessed on 21 April 2025) [13]. The analysis was focused on the ATP-binding domain (Pfam: PF00005, ABC_tran) and transmembrane domains such as ABC2_membrane_3 (PF12698). The 16 proteins that contained both an ATP-binding domain and at least one transmembrane domain were selected as strong candidates for further study (Supplementary Materials, Table S4). For consistent nomenclature, the final set of paralogs derived from potato genome was systematically renamed as *StABCG25-1* through *StABCG25-6*. This assignment was recorded and verified using annotation information from the working file, Stuberosum_448_v4.03.P14.annotation_info, downloaded from Phytozome [12], which integrated best BLAST hits and functional domain data from the initial screening steps. Furthermore, the genes identified in the co-expression networks (WGCNA) of the *StABCG25* candidates were also annotated and verified against this same annotation file to ensure a unified genomic context throughout the study.

### 2.2. Promoter Analysis for ABA-Responsive Elements

For each of the 16 selected candidates, a 2000 bp promoter sequence upstream of the gene was downloaded from the *Solanum tuberosum* genome (v4.03 assembly) in Phytozome [12]. The sequences were prepared by trimming any leading ‘N’ bases and were padded to ensure a uniform length of 2000 nt for the analysis. The prepared promoter sequences were submitted to the PlantCARE database (https://bioinformatics.psb.ugent.be/webtools/plantcare/html/, accessed on 23 April 2025) to find putative regulatory elements [14]. A scoring system named “ABA Score” was developed to identify genes most likely responsive to ABA. The selection of *cis*-regulatory elements for this score was based on their well-documented roles in ABA and stress signaling. These motifs were categorized into four major functional groups to ensure comprehensive coverage of the ABA regulatory network:

**The Abscisic Acid Responsive Element (ABRE) family:** This group includes ABRE, ABRE4, and ABRE3a. These are all known functional variations of the core ABRE motif, sharing a similar ACGT core sequence, and represent the primary ABA-responsive elements [15].

**MYB and MYC transcription factor binding sites:** This category encompasses numerous synonymous annotations (e.g., MBS, MYB, Myc, Myb-binding site) for the DNA binding sites of the large MYB and MYC transcription factor families. These factors are key downstream regulators in the ABA signaling pathway [16].

**Jasmonate-coupled elements:** The motifs CGTCA-motif and TGACG-motif were included due to the well-documented crosstalk between the ABA and jasmonic acid (JA) signaling pathways, making their presence significant in ABA-responsive promoters [17].

**The RY-element:** This specific motif was included for its established role in ABA-regulated processes, particularly during seed development [18]. To quantify the overall density of these potential regulatory sites, the ABA Score was calculated as a cumulative sum of every individual motif instance identified within a promoter. The score was calculated according to the formula below:

**ABA Score** = ABRE + ABRE4 + ABRE3a + CGTCA-motif + MBS + MYB + MYC + MYB recognition site + MYB-like sequence + Myb + Myb-binding site + Myc + RY-element + TGACG-motif (Supplementary Materials, Table S5). Finally, candidates with an ABA Score of 8 or higher were selected for further analyses (Table 1).

### 2.3. RNA-Seq Data Analysis

In this study, an in silico transcriptomic analysis was performed to investigate the molecular responses of potato under drought stress. The primary dataset was obtained from the NCBI Sequence Read Archive (SRA: SRP451490; BioProject: PRJNA998742) and consists of 150 bp paired-end Illumina reads generated from tuber tissues of the potato cultivar Toyoshiro, developed by Calbee, Inc. (Utsunomiya, Tochigi, Japan), under controlled water-deficit conditions. Previous reports indicate that Toyoshiro is susceptible to drought, showing significant yield reduction under water-limited environments [19]. For comparative and validation purposes, an additional dataset described by Barra et al. [20] (GEO accession: GSE140083) was incorporated. This dataset includes transcriptomic profiles of two potato cultivars with contrasting drought tolerance: the highly tolerant Clone 37 FB and the highly susceptible Cardinal and consisted of 126 bp paired-end Illumina reads generated from leaf tissues of the potato.

#### 2.3.1. Data Pre-Processing and Quality Control

Raw RNA-Seq reads for both tuber and leaf tissues were obtained from the Sequence Read Archive (SRA) [21] and analyzed on the Galaxy platform [22]. The faster download and extraction of SRA reads were made using Faster Download and Extract Reads in FASTQ (Version 3.1.1) in Galaxy [21]. An initial quality control was performed to assess the overall profile of the raw data. The quality control was performed using Falco (Version 1.2.4) [23] and the results were summarized with MultiQC (Version 1.27) [24]. Based on this assessment, a pre-processing step was applied to clean the data, with tools selected to best address the specific quality profile of each dataset. The tuber dataset, which exhibited significant adapter contamination and high read duplication rates, was processed using Fastp (Version 1.0.1) [25], an all-in-one tool suited for comprehensive cleaning. In contrast, the higher-quality leaf dataset required only standard trimming, for which Cutadapt (Version 5.1) [26] was employed to remove residual adapters and filter reads below a minimum length of 50 bp. For both datasets, reads that were too short or of poor quality after trimming were discarded to ensure the integrity of the downstream analysis.

#### 2.3.2. Alignment to the Reference Genome

The processed, high-quality reads were aligned to the *Solanum tuberosum* reference genome (version SolTub_3.0, obtained from Ensembl Plants) using the STAR (Spliced Transcripts Alignment to a Reference) aligner (Version 2.7.11b) [27]. The required files for STAR tool were downloaded from Ensembl Plants Release 60 [28]. The alignment was performed in paired-end mode. Post-alignment statistics, obtained via MultiQC (Version 1.27) [24], confirmed high overall mapping rates for both experiments, with a large proportion of reads mapping uniquely to the genome, indicating successful alignment.

#### 2.3.3. Gene Expression Quantification

Prior to quantification, the library strandedness was determined using the infer_experiment.py script (from the RSeQC package v5.0.3) [29]. The results confirmed that both the tuber and leaf libraries were unstranded. Subsequently, gene-level read counts were generated from the aligned BAM files using the corresponding gene annotation (GTF) file. For the tuber analysis, counts were generated using STAR’s internal quantMode GeneCounts function [27]. For the leaf analysis, featureCounts (Version 2.1.1) [30] was used. In both cases, the counting was performed in unstranded mode, and for featureCounts, a minimum mapping quality (MAPQ) filter was applied to exclude ambiguous alignments. The resulting count matrices, representing the expression level of each gene across all samples, were used as input for subsequent differential gene expression analysis with DESeq2 (Version 2.11.40.8).

#### 2.3.4. Z-Score Transformation for Heatmap Visualization

The heatmap displays gene-wise Z-scores for the candidate ABCG25 transcripts to visualize relative expression patterns. To generate these values, the count data was first normalized using the variance-stabilizing transformation (VST) function from the DESeq2 package [31]. Subsequently, the VST-normalized value for each gene was converted to a Z-score by subtracting the mean and dividing by the standard deviation across all samples. This scaling method highlights relative expression changes, preventing genes with high absolute expression from dominating the visualization. The final heatmap was generated using TBtools-II (version 2.112) [32].

### 2.4. Weighted Gene Co-Expression Network Analysis (WGCNA)

The WGCNA was used to identify gene modules in the tuber (Toyoshiro) and leaf (FB clone) datasets and to study how they are related to drought stress. The analysis was performed in R (Version 4.4.2) [33] with the RStudio interface (Version 2024.12.0) [34], using the WGCNA R package (Version 1.7) [35].

#### 2.4.1. Data Input and Pre-Processing

For the analysis, we used variance-stabilized (VST) gene expression values from the DESeq2 package [31]. To reduce noise and calculation time, the genes with the lowest variance (bottom 10%) were filtered out. However, the ABCG25 transcript, which was important in the differential expression analysis, was kept in both datasets even if its variance was low. To check sample quality, clustering analysis was performed, and no outlier samples were found.

#### 2.4.2. Network Construction and Module Detection

A signed co-expression network was built following the standard WGCNA workflow. Choosing the correct soft-thresholding power (β) was a key step to approximate a “scale-free topology.” We tested β values from 1 to 20. For the tuber (Toyoshiro) dataset, the best soft-thresholding power was β = 13, which gave a truncated R^2^ of 0.987 with reasonable average connectivity. The same procedure in the leaf (FB clone) dataset also resulted in β = 13, yielding a truncated R^2^ of 0.893. With these powers, adjacency matrices were then created and converted into Topological Overlap Matrices (TOM). Using TOM, hierarchical clustering trees were built, and gene modules were identified with the dynamic tree cut method (minimum module size = 30 genes). Similar modules (eigengene correlation < 0.25) were later merged.

#### 2.4.3. Module-Trait Association and Hub Gene Identification

To study how the modules relate to experimental traits, we calculated module eigengenes (MEs), which represent the main expression pattern of each module. These MEs were then correlated with drought stress and control conditions. Modules that showed a strong and significant correlation with drought stress were considered biologically important. Within these modules, hub genes were identified based on high module membership (kME), meaning strong connections to the module’s expression profile.

#### 2.4.4. Network Visualization

For visualization, the co-expression networks were exported to Cytoscape (Version 3.10.2) [36]. To simplify the networks, strict weight filters were applied: edges above the 99.7th percentile of all connections were kept for the tuber (Toyoshiro) dataset, and edges above the 99.6th percentile were kept for the leaf (FB clone) dataset. These thresholds helped highlight the hub genes and their strongest interactions within the drought-related modules.

#### 2.4.5. Differential Alternative Splicing (AS) Analysis

Differential alternative splicing (AS) events in leaf and tuber tissues were analyzed using rMATS-turbo (v4.1.2) [37]. The input data included BAM files aligned with STAR [27] (Dobin et al., 2013) and the *Solanum tuberosum* (v3.0.61) gene annotation file. The analysis was performed with unstranded, 150 bp paired-end reads, and five main AS types were studied: Skipped Exon (SE), Retained Intron (RI), Mutually Exclusive Exons (MXE), Alternative 5′ Splice Site (A5SS), and Alternative 3′ Splice Site (A3SS). Significant AS events were defined using two thresholds: False Discovery Rate (FDR) < 0.05 and absolute Percent Spliced In difference (|ΔΨ|) > 0.1. The list of significant events was then checked to see if target genes, including *ABCG25*, showed differential splicing.

#### 2.4.6. Ontology Analysis

Gene lists defined for WGCNA modules (turquoise_genes.txt, plum_genes.txt, orangered1_genes.txt, lightblue3_genes.txt) were tested against all network genes (universe_all_nodes.txt) as the background set. Functional annotations were based on the *Solanum tuberosum* genome (v4.03) [38] and the corresponding GO mapping file (goterms.txt) downloaded from Ensembl Plants [28].

GO enrichment was performed with the clusterProfiler package (v4.8.1) using a hypergeometric test [39]. *p*-values were adjusted by the Benjamini–Hochberg method, and significance was set at q ≤ 0.05 [40]. Gene sets smaller than 5 or larger than 5000 were excluded. For each module, significantly enriched GO terms were identified, fold enrichment values were calculated, and biological processes were compared across modules [41,42].

### 2.5. Deep Learning-Based Protein Structure Prediction (AlphaFold2–ColabFold)

In this study, the prediction of the three-dimensional (3D) structures of proteins (listed in Table 1) was carried out using AlphaFold2, a deep learning-based artificial intelligence system selected for its proven accuracy in predicting protein structures directly from amino acid sequences [43]. To this end, we employed the ColabFold version of AlphaFold (Version 2), an open-source implementation accessible via GitHub (https://github.com/sokrypton/ColabFold, accessed on 16 July 2025), and conducted all modeling procedures on the Google Colab platform (https://colab.research.google.com/github/sokrypton/ColabFold/blob/main/AlphaFold2.ipynb, accessed on 16 July 2025). FASTA-formatted sequences were uploaded directly to the ColabFold interface. The platform automatically generated multiple sequence alignments (MSAs) using MMseqs2 (Version AlphaFold 2.0), relying on its default settings. Based on these alignments, AlphaFold (Version 2.0) carried out the 3D structure predictions. The resulting models were evaluated using the quality metrics provided by ColabFold (Version AlphaFold 2.0) for visual inspection and preliminary analysis. The predicted 3D structures were examined in PyMOL (Version 3.10) [44] and UCSF Chimera (Version 1.19) [45]. Before initiating calculations, all models were first processed using the pdb2amber tool (Version Amber 22) to eliminate structural inconsistencies. This step ensured more reliable parameter file generation in the subsequent tleap process. In addition, prior to the heating and production stages of the simulations, all models underwent a separate energy minimization step using OpenMM (Version 7.0) [46]. This optimization allowed the systems to start with lower initial potential energy. As a result, the computational cost was reduced, and longer MD simulations could be performed more efficiently.

### 2.6. Molecular Docking Processes

To prepare the predicted protein models for docking, the prepare_receptor4.py script from MGL Tools (Version 1.5.7) was used [47,48]. PDB files were converted into PDBQT format after the assignment of Gasteiger charges. For each protein, grid box dimensions were determined using a custom Python (Version 3.13.1) script named prepare_box.py (Version 1.0), which we developed and made publicly available via GitHub (Supplementary Materials, Sheet: GitHub Archive of Docking Inputs and Analysis Scripts). This script scans all proteins within a given directory, extracts the minimum and maximum x, y, and z atomic coordinates, and calculates the box center as the average of these extremes. The script then outputs AutoDock Vina (Version 1.1.2 Linux x86)-compatible command lines, including the box sizes (size_x, size_y, size_z) and center coordinates (x, y, z).

The ligand was retrieved from the ChemSpider database [49], energy-minimized using Amber [50], and converted into docking-ready PDBQT format using the prepare_ligand4.py script within MGL Tools (Version 1.5.7) [47,48], with Kollman charges assigned. Docking simulations were performed using vina.exe. The resulting output files were processed using vina_split.exe to extract individual poses, and the top-scoring complexes were visualized using Discovery Studio Visualizer (DSV) (Version 2024) [51].

### 2.7. Molecular Dynamics Simulations

Following the docking procedure, the required simulation input files were generated using the pdb4amber and tleap programs (Version Amber 24). Molecular dynamics simulations were then initiated using the OpenMM (Version 7.0) software package [46] on Google Colab, utilizing NVIDIA Tesla A100 80 GB GPUs. To begin, the ligand file obtained after docking (via vina_split.exe) was converted from PDBQT to PDB format. This file was then opened in PyMOL (Version 3.10) [44], where hydrogens were added using the h_add command, and the file was saved as ligand_H.mol2. In the next step, the antechamber module in AMBER (Version 24) was used to assign RESP charges and convert the ligand to a mol2 file with atom types compatible with the GAFF force field, using the following command:

antechamber -i ligand_H.mol2 -fi mol2 -o ligand_charge.mol2 -fo mol2 -at gaff -dr no -c resp -nc 0

Subsequently, missing force field parameters were resolved using the parmchk (Version 2 (Amber 24)) tool, which generated a corresponding FRCMOD file (ligand.frcmod). In the following stage, the tleap program within AMBER (Version 24) was launched. The ff19SB force field was loaded for the protein, and the ligand.frcmod file was used for the ligand. Topology and coordinate files for the complex were then generated. The combine command was used to merge the protein and ligand into a single complex, and set default PBRadii mbondi2 was applied to select a radius model suitable for MM/PBSA calculations. Next, to prepare the system for explicit solvent simulations, the leaprc.water.opc file was sourced and the complex was solvated in a cubic OPC water box with a 10 Å buffer distance. The use of the OPC water model in this study is based on the recommendation from the Amber24 Manual [52], which highlights that although TIP3P is computationally efficient, OPC and OPC3 models offer better electrostatic accuracy. Specifically, the four-point OPC model has been shown to reproduce experimental properties such as density and diffusion coefficients with high accuracy. This recommendation aligns well with earlier studies in the literature [53,54,55]. Force field parameters for the ligand were loaded from the ligand.frcmod file, and solvated system topology and coordinate files were generated using the command:

saveamberparm complex complex_su.prmtop complex_su.crd

In order to allow stable simulations with an extended time step of 4 fs, the prmtop file was further processed using the hmassrepartition command of the ParmEd (Version 4.3.0) program [56]. This step redistributes mass by increasing hydrogen atom masses while reducing the mass of the bonded heavy atoms accordingly, preserving the total atomic mass. As a result, simulations can be safely run with a longer time step (4 fs instead of 2 fs) without compromising the physical accuracy of the system, enabling more efficient use of computational resources and time.

Following these steps, the complex_su.prmtop and complex_su.crd files generated separately for each protein were transferred to the Google Colab environment. To initiate the simulations, a Python script was written by us based on the OpenMM (Version 7.0) user guide [57]. This script is available at: https://github.com/bkurt00/ABCG25_md/blob/main/start_md.py (accessed on 30 September 2025). Using our custom script, energy minimization was performed first, followed by a stepwise heating protocol from 0 K to 300 K in 50 K increments. This protocol ensured smooth thermalization without introducing structural instabilities, as confirmed by subsequent RMSD analysis. At each temperature step, 5000 simulation steps were run. During this process, the system was maintained under constant volume (NVT) conditions using Langevin dynamics (300 K, 1 ps^−1^ friction coefficient). Throughout the production phase, coordinate outputs were recorded every 0.1 ns in NetCDF format and saved, along with checkpoint files, into the nc directory. The simulations proceeded without interruption and the production phase was extended up to 500 ns. Subsequently, energy calculations were performed, followed by RMSD and RMSF analyses using CPPTRAJ (Version V6.18.1 (AmberTools)). All simulation parameters and stepwise details are summarized in Supplementary Materials, Table S7.

### 2.8. MM/GBSA and MM/PBSA Free Energy Calculations

To evaluate the binding affinities of the ligands to their respective protein targets, MM/GBSA and MM/PBSA methods were employed [58]. These methods offer a semi-empirical approach to estimate molecular mechanics (MM) energies and the binding free energies (ΔG_bind) in solution. The binding free energy is calculated using the following expression:

ΔG_Bind_ = G_complex_ − (G_receptor_ + G_ligand_)

Here, the total free energy (G) of each component is defined as [59]:

G = E_mm_ + G_solv_ − TS

In this equation, Emm includes bonded, electrostatic, and van der Waals interactions, while Gsolv accounts for solvation free energy, which comprises both polar (via GB or PB models) and nonpolar contributions. T denotes the absolute temperature in Kelvin, and S refers to the system’s entropy.

In the present study, MM/GBSA and MM/PBSA approaches were adopted to estimate the binding free energies, primarily focusing on enthalpic contributions. All calculations were carried out using the MMPBSA.py module from the AMBER (Version 24) software suite [58]. For preprocessing and generating the required topology files, the ante-MMPBSA.py utility was used. During the analyses, ions and solvent molecules were excluded to focus solely on the protein–ligand interactions. Reference ligand residues within the binding pockets were defined individually for each complex.

For the energy calculations, the full set of trajectory frames obtained from molecular dynamics simulations was included. Both Generalized Born (GB, with igb = 5) and Poisson–Boltzmann (PB) models were applied under 0.15 M ionic strength conditions. All input parameters and in configuration files used for the calculations are provided as links in Supplementary Materials. (Sheet: GitHub Archive of Docking Inputs and Analysis Scripts).

## 3. Results

### 3.1. Contrasting Drought Responses of the StABCG25 Gene Family in Tolerant and Susceptible Potato Cultivars

**Figure 1:**
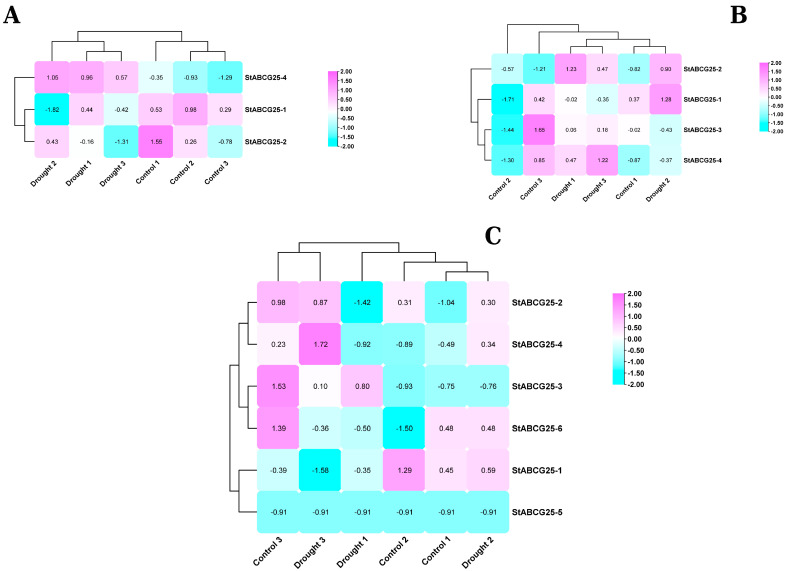
Drought stress induces distinct expression patterns of *StABCG25* genes in tolerant and susceptible potato cultivars. Hierarchical clustering and heatmaps of gene-wise Z-scores for *StABCG25* transcripts in (**A**) susceptible Toyoshiro, (**B**) tolerant FB Clone, and (**C**) susceptible Cardinal cultivars under control and drought conditions. Expression values were derived from VST-normalized counts. The color key indicates upregulation (magenta) and downregulation (cyan). The analysis reveals a coordinated upregulation of *StABCG25* transcripts in the tolerant FB cultivar, contrasting with a general downregulation in Cardinal and a conflicting expression pattern in Toyoshiro.

The response of the *StABCG25* gene family to drought stress exhibited clear differences depending on the tolerance level of potato cultivars (Figure 1). In the drought-tolerant FB cultivar, key isoforms such as *StABCG25-2* and *StABCG25-4* were consistently activated under stress, whereas in the susceptible Cardinal cultivar most of these genes were repressed. Interestingly, the susceptible Toyoshiro cultivar displayed a contradictory profile; while *StABCG25-4* was activated, *StABCG25-2* was suppressed. These findings suggest that drought tolerance in potato depends on the coordinated and consistent activation of *StABCG25* genes under stress conditions. The tolerant FB cultivar maintains this coordination, while the susceptible cultivars either show general repression (Cardinal) or a dysfunctional response (Toyoshiro), ultimately failing in this defense mechanism.

### 3.2. The Effect of Drought Stress on Alternative Splicing in the Potato Transcriptome

To understand how drought stress affects post-transcriptional regulation in potato, we performed a genome-wide alternative splicing (AS) analysis using RNA-Seq data from leaf and tuber tissues. The analysis was carried out with the rMATS-turbo tool, using statistical cutoffs of FDR < 0.05 and |ΔPSI| > 0.1 to identify significant splicing events. According to the results, 111 significant AS events were found in the leaf tissue of the Cardinal variety, 379 events in the leaf tissue of the FB clone, and 345 events in the tuber tissue. These findings show that drought stress triggers a strong and tissue/genotype-specific splicing response in potato. The details are presented in Table 2.

Among all AS types, retained intron (RI) was the most common event in all tissues. The splicing patterns also changed depending on the variety. In the FB clone, the number of RI (212) and A3SS (72) events was much higher than in the Cardinal variety, where only 46 RI and 42 A3SS events were observed. In tuber tissue, RI (170) and SE (80) were the most frequent types. These results suggest that the splicing response to drought stress is both tissue-specific and genotype-dependent. We also examined specific genes related to drought response. One of them, *ABCG25* (gene ID: PGSC0003DMT400004973), is known to play an important role in drought stress signaling. However, our rMATS analysis did not detect any significant splicing events in ABCG25 in either leaf or tuber tissues.

In conclusion, drought stress in potato triggers a wide and complex alternative splicing program, especially affecting retained introns and splice site choices. However, some key genes like *ABCG25* may respond mainly through transcriptional regulation, without changes in their splicing patterns. This shows that plants use multiple levels of regulation to adapt to environmental stress, and alternative splicing is one of the important layers, but not the only one.

### 3.3. Co-Expression Network Analysis of StABCG25 Genes in Leaf Tissue

**Figure 2:**
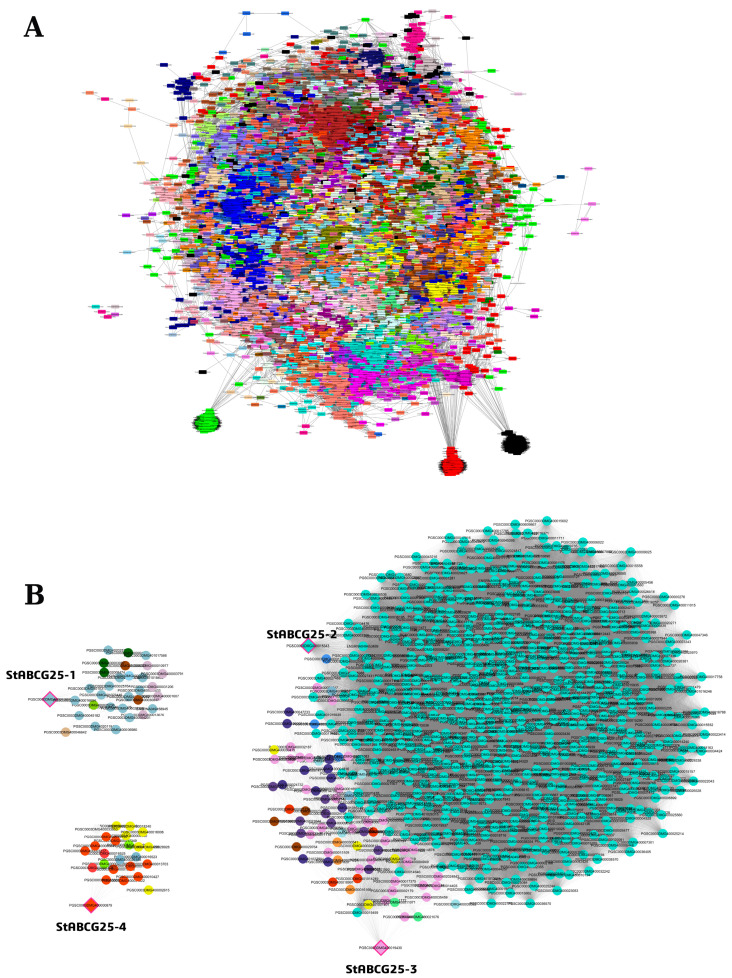
Gene co-expression network (WGCNA) in a drought-resistant potato variety in leaf tissue (**A**). Nodes represent genes, with colors indicating their assigned module. The figure highlights the distinct network roles of the *StABCG25* family: the hub gene *StABCG25-2* (turquoise), the peripheral *StABCG25-1* (lightblue3), and the negatively correlated *StABCG25-4* (orangered1) and *StABCG25-3* (plum) (**B**). Full gene lists, module assignments, and edge weights are provided in Supplementary Materials, Tables S8–S10.

To investigate the transcriptional landscape and potential functional roles of *StABCG25* genes in leaf tissue, a WGCNA was performed (Supplementary Materials, Tables S8–S10). The analysis identified several distinct gene modules, with turquoise, lightblue3, orangered1, plum, mediumpurple, and yellow2 emerging as prominent clusters of co-expressed genes (Figure 2A,B). We refined module assignments by considering both primary module colors and bestKMEmodule predictions.

#### 3.3.1. Network Topology and Functional Context of *StABCG25-1*

The co-expression network analysis for *StABCG25-1* examined 31 genes, which predominantly clustered into the largest module, lightblue3 (18 genes), along with thistle3 (5 genes) and darkgreen (3 genes) (Supplementary Materials, Table S8). The *StABCG25-1* gene itself exhibited a very strong positive correlation (r = 0.8882) with the eigengene of its assigned lightblue3 module, indicating positive regulation. Its high kTotal value (~697.06) suggests a significant central role in the module’s core functioning. Although no genes were classified as Hub genes, the darkgreen module displayed the highest mean intramodular connectivity (kWithin ~106.74), representing the most tightly co-expressed cluster. The validity of the WGCNA module structure is supported by a moderate-to-strong positive correlation (r = 0.6640) between Module Membership (kME) and Total Connectivity (kTotal). Functionally, the largest lightblue3 module members include E3 Ubiquitin Ligases and DNA Repair proteins, while the thistle3 module is associated with Auxin Signaling and Calcium/Lipid Binding. The most tightly connected darkgreen module is focused on secondary metabolism via 2-Oxoglutarate Oxygenase and regulatory mechanisms involving a Transcriptional Regulator (SIN3-LIKE).

#### 3.3.2. Critical Role of *StABCG25-2* and *StABCG25-3* in Stress and Defense Networks

The general genomic network analysis revealed that approximately 50% of the genes clustered in the turquoise, blue, and brown modules, which represent the most dominant structures in the network (Supplementary Materials, Table S9). Notably, *StABCG25-2* and *StABCG25-3* genes were assigned to the brown module, which exhibits the strongest intramodular connection density (kWithin = 0.490). Both genes exhibited a very strong positive correlation with the module’s central hub genes (bestKMEValue values ranging from ~0.84 to ~86), and their notably high intra-module centrality values (~0.835) confirm their critical role in the module’s main functional activities. Examination of the regulatory content of the brown module showed a prevalence of Serine/threonine-protein kinases, which are central to signal transduction, alongside C3H and C2H2 zinc finger TF families often linked to development and stress responses. At the functional level, genes within the brown module were associated with key pathways, including Heat Shock Proteins, Glutathione S-transferases, and disease resistance proteins. This finding substantiates the hypothesis that the *StABCG25* genes act as critical transporters or regulators within the plant’s detoxification and general defense response network against abiotic and biotic stress factors. Other dominant modules, turquoise, focused on disease resistance and auxin responses, while blue concentrated on transferases and DNA binding proteins, indicating that the system encompasses various fundamental physiological processes.

#### 3.3.3. *StABCG25-4* Network: Loose Topology and Regulatory Gene Enrichment

WGCNA and functional analyses applied to the smaller 25-gene dataset for *StABCG25-4* indicated that the majority of genes (76%) clustered into three dominant modules: orangered1 (11 genes), yellow2 (7 genes), and brown1 (2 genes) (Supplementary Materials, Table S10). Centrality analysis revealed a loose module structure, as all genes were labeled Non-Hub. The Mean Out-Module Centrality (kOut ~ 589.66) was approximately 12-fold higher than the In-Module Centrality (kWithin ~ 48.96), and the severely negative Centrality Difference (kDiff ~ 540.7) indicates that genes were far more connected to the overall network than to their respective modules, suggesting weak module boundaries. Functionally, these dominant modules are rich in key regulatory elements, with the identification of five Transcription Factors (TFs) and one Kinase (RLK-Pelle_WAK receptor-like kinase) belonging to significant TF families such as NAC, WRKY, AP2/ERF, and NF-YC. The most frequent annotation among gene names in these dominant modules was AVR9_CF-9 RAPIDLY ELICITED PROTEIN, associated with defense and signaling pathways. Within this structure, the most central genes, representing the strongest expression pattern of their respective modules, were identified: ZINC FINGER PROTEIN ZAT10 (kME = 0.9909) in orangered1, RING_U-BOX SUPERFAMILY PROTEIN (kME = 0.9637) in yellow2, and PROTEIN TRANSPORT PROTEIN SEC23B (kME = −0.9575) in brown1.

#### 3.3.4. Gene Ontology Enrichment and Functional Analysis in Leaf Tissue 

To infer the biological functions associated with the *StABCG25* genes and their co-expressed partners, a Gene Ontology (GO) enrichment analysis was performed on the identified modules. No GO terms remained statistically significant after the application of a strict False Discovery Rate (FDR) correction for multiple testing. However, an exploratory analysis conducted at a less stringent threshold (raw *p*-value < 0.05) provided insights into potential biological processes relevant to drought adaptation (Supplementary Materials, Table S11). The turquoise module, containing the central *StABCG25-2* gene, was associated with hydrolase activity (GO:0004553), carbohydrate metabolism (GO:0005975), monooxygenase activity (GO:0004497), and protein ubiquitination (GO:0016567). Other modules were linked to transferase activity (lightblue3; GO:0016740), glycosyltransferase activity (mediumpurple; GO:0016757), embryo sac development (orangered1; GO:0009553), and DNA repair (yellow2; GO:0006281). Collectively, these findings embed the *StABCG25* family within a complex regulatory network linked to metabolic processes, protein modification, developmental regulation, and cellular stress responses.

### 3.4. Co-Expression Network Analysis of StABCG25 Genes in Tuber Tissue

**Figure 3:**
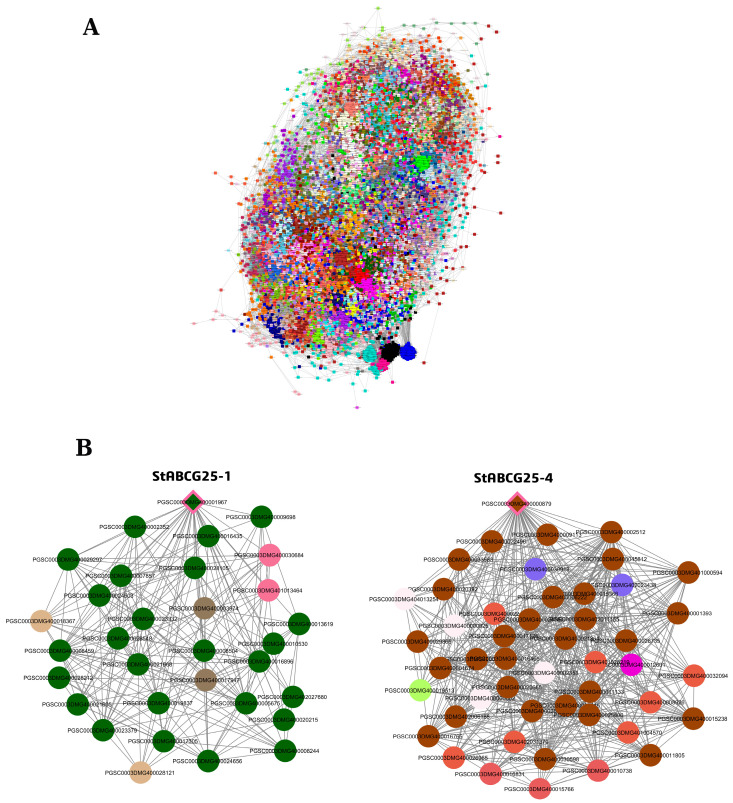
Gene co-expression network in tuber tissue and the positions of *StABCG25* genes within their modules (see Supplementary Materials, Tables S12 and S13 for full gene lists and connectivity values). (**A**) The co-expression network constructed from all genes. Each color represents a different module. (**B**) First-degree neighbors of the drought-sensitive variety’s co-expressed genes *StABCG25-1* (darkgreen module, left) and *StABCG25-4* (chocolate4 module, right) within their respective modules. Diamond-shaped nodes represent *StABCG25* genes, while circles represent other co-expressed genes. The colors are consistent with the module colors shown in panel (**A**), and the edges (lines) represent connections between genes.

To investigate the transcriptional landscape and distinct functional roles of the *StABCG25* family members in the tuber tissue of a drought-sensitive variety, a Weighted Gene Co-expression Network Analysis (WGCNA) was performed (Figure 3). The analysis revealed highly integrated regulatory networks that utilize complex interaction strategies, particularly around *StABCG25-1* and *StABCG25-4* genes, in response to stress (Supplementary Materials, Tables S12 and S13).

#### 3.4.1. Network Topology and Regulatory Strategy of *StABCG25-1*

The co-expression analysis focusing on *StABCG25-1* examined 32 genes, the vast majority of which (~81% or 26 genes) clustered into the dominant darkgreen module (Supplementary Materials, Table S12). This concentration suggests a highly cohesive and tightly regulated expression program within the tuber.

The network architecture indicates that genes function primarily as critical network connectors rather than internal module hubs. Centrality analyses revealed that Out-Module Connectivity (kOut) was significantly dominant over In-Module Connectivity (kWithin), as evidenced by the extremely negative average Centrality Difference (kDiff mean: −497.134). The *StABCG25-1* gene itself reflects this structure, showing a strong positive correlation (kME = 0.849) with its eigengene but a high kOut value (kDiff = −478.3), emphasizing its role as a key linking node in the overall network. The darkgreen module is tightly regulated, centered around the *PGSC0003DMG400028548* gene, which exhibits the strongest positive correlation with the eigengene (kME = 0.93869).

A key regulatory feature is the presence of four dominant regulatory genes: two Transcription Factors (NAC and RWP-RK), one Regulator, and one Kinase (Receptor-like Kinase 17). Intriguingly, two of these four regulators (one TF and the Kinase) show a strong negative correlation (kME values of −0.822 and −0.858) with the module’s primary expression pattern and *StABCG25-1*. This inverse expression profile suggests that these genes may act as potential repressors or represent an antagonistic regulatory pathway. Functionally, the genes in the darkgreen module are diverse, encompassing ABC Transporters, Chloride Channels, and Protein Phosphatases, confirming the comprehensive integration of *StABCG25-1* into multiple cellular functions relevant to drought response.

#### 3.4.2. *StABCG25-4* Network: Density Differences and Regulatory Mechanisms

The WGCNA focusing on *StABCG25-4* (analyzed in root tissue but compared here to the tuber environment) highlights structural and functional differences across tissues (Supplementary Materials, Table S13). Similar to the *StABCG25-1* network, the *StABCG25-4* root network exhibits an extremely negative average kDiff value (not specified here, but inferred from the text), confirming the dominant role of genes as critical network connectors interacting intensively with other modules.

However, the *StABCG25-1* tuber network (darkgreen module) is significantly more dense and internally connected (kWithin mean: ~72.6 and 81% gene concentration) than the *StABCG25-4* root network (chocolate4 module, kWithin mean: ~38.15 and 62.5% gene concentration), which appears more diffuse and flexible. Both *StABCG25-1* (kME = 0.849) and *StABCG25-4* (kME = 0.873) hold central, positively correlated roles in their respective dominant modules.

A shared dual-acting regulatory strategy is observed, as both dominant modules contain Kinases and Transcription Factors that exhibit a strong negative correlation with the module’s main expression profile. A critical structural difference in the *StABCG25-4* network is that the gene with the absolute highest kME value (0.954) is located in a sub-module outside the dominant chocolate4 cluster, suggesting that the most centralized function in the root network is concentrated within a compact, separate co-expression group.

#### 3.4.3. Gene Ontology Enrichment and Biological Insights in Tuber Tissue 

To infer the biological functions associated with the *StABCG25* genes and their co-expressed partners in tuber tissue, a Gene Ontology (GO) enrichment analysis was performed (Supplementary Materials, Table S14). No GO terms remained statistically significant after the application of a strict False Discovery Rate (FDR) correction for multiple testing. However, an exploratory analysis conducted at a less stringent threshold (raw *p*-value < 0.05) provided insights into potential biological processes relevant to stress adaptation (Supplementary Materials, Table S14):

- The darkgreen module, which includes *StABCG25-1*, was associated with protein homodimerization activity (GO:0042803). This suggests that *StABCG25-1* may function through protein–protein interactions as part of larger regulatory complexes.
- The chocolate4 module (*StABCG25-4*) was enriched in processes related to plastid transcription (GO:0042793) and plastids (GO:0009536) in general, pointing to a potential role for *StABCG25-4* in plastid-related signaling or functions.
- Other modules, such as navajowhite4, reflected core stress and regulatory mechanisms by being enriched in terms of cold acclimation (GO:0009631), chromatin remodeling (GO:0006338), and the regulation of transcription by RNA polymerase II (GO:0006357).

Collectively, these results show that the *StABCG25* genes in the tuber tissue of the drought-sensitive variety are embedded in a complex regulatory network that involves protein interactions, plastid functions, and the transcriptional reprogramming of gene expression essential for abiotic stress response.

### 3.5. AlphaFold2 Modeling Quality Assessment

The 3D structures of the proteins under investigation were predicted using AlphaFold2, and the resulting models were evaluated based on three quality metrics provided by the algorithm [43]:

1. pLDDT (Predicted Local Distance Difference Test): A confidence score assigned to each amino acid position on a scale from 0 to 100, reflecting the local reliability of the model. Scores above 90 indicate high confidence in the predicted region.
2. PAE (Predicted Aligned Error): This metric reflects the positional uncertainty between different regions of the protein, primarily used to assess inter-domain alignment reliability. Low PAE values (<5 Å) suggest well-aligned and structurally consistent relationships between domains.
3. Sequence Coverage: Represents the depth of evolutionary information (homologous sequences) included in the model. High sequence coverage implies the prediction is supported by rich sequence data and is thus considered more reliable.

The graphical outputs for these three metrics, generated by AlphaFold (Version 2) for each model, are provided in Supplementary Materials, Figures S1–S7. A summary of the model-specific evaluations is presented in Table 3. Overall, all models showed high sequence coverage, and intra-domain structural confidence (PAE intra) was generally strong across the dataset. Although certain models—particularly *StABCG25-5a* and *StABCG25-6*—displayed increased uncertainty in inter-domain positioning (PAE inter), the average pLDDT scores for all models remained within acceptable limits. Among them, *StABCG25-1* and *StABCG25-4* stood out as having the highest structural confidence.

### 3.6. Docking and Molecular Dynamics Simulation Analyses

**Figure 4:**
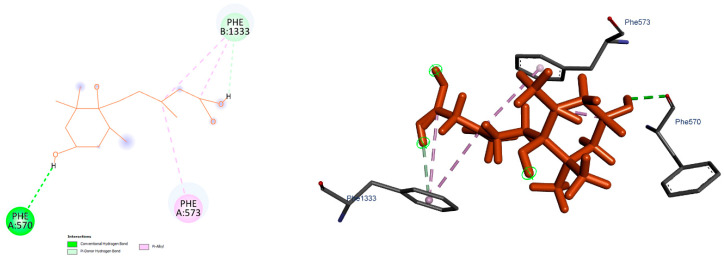
Figure showing 2D and 3D representation of binding modes for Model 5B.

To explore the potential interactions between the ligand and the target protein, molecular docking studies were performed. The resulting binding scores across models ranged from −5.9 to −6.6 kcal/mol. Among them, Model 5b displayed the lowest binding energy (−6.6 kcal/mol), suggesting it represents the most stable binding mode compared to the others (Table 4). Three-dimensional visual inspection of the binding poses indicated that the ligand generally settled into a similar region within the binding pocket across all models. However, conformational variations were observed, particularly in the flexible regions of the ligand. These differences led to changes in hydrogen bond positioning and, in some cases, an increase in hydrophobic contacts. Such observations suggest that the ligand may adopt multiple stable conformations within the binding region. The most stable interaction profile, corresponding to the *StABCG25-5b* model, is illustrated in Figure 4. The remaining docking poses are presented in Supplementary Materials, Figures S8–S14. The overall similarity of docking scores supports the idea that the ligand can engage in several consistent yet distinct interaction modes within the binding site.

### 3.7. RMSD Analysis

**Figure 5:**
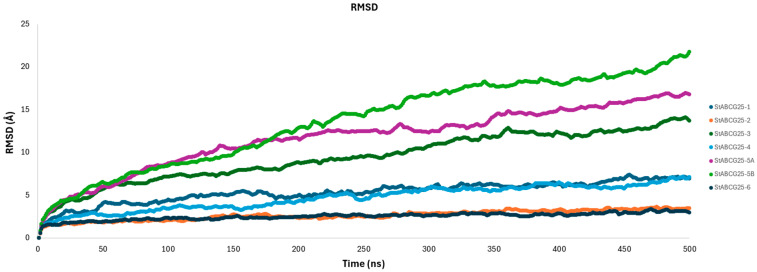
RMSD Analysis of Model Proteins.

To evaluate the structural stability of each model during molecular dynamics simulations, root-mean-square deviation (RMSD) analyses were conducted. The resulting RMSD plots are presented in Figure 5. Below is a separate evaluation of each model’s RMSD trajectory:

- The *StABCG25-1* model displayed a continuous increase up to approximately 450 ns, eventually reaching ~7 Å by the end of the simulation, indicating a considerable structural deviation. However, the RMSD curve started to plateau after 450 ns, suggesting a transition toward a more stable conformation.
- The *StABCG25-2* model showed the lowest RMSD values among all models, maintaining a stable trajectory around 3.5 Å throughout the simulation. This consistency indicates that the model remained close to its initial conformation and exhibits strong structural stability.
- The *StABCG25-3* model demonstrated a marked RMSD increase during the first 400 ns, after which the curve gradually plateaued around 14 Å. This pattern suggests an initial structural adaptation phase, followed by convergence to a more stable form. It appears that the simulation time was sufficient for this model to reach a near-final conformation.
- The *StABCG25-4* model exhibited a steadily increasing RMSD with moderate fluctuations, stabilizing around 7 Å toward the end of the simulation. Despite undergoing some structural rearrangements, the model overall maintained a reasonably balanced structure.
- The *StABCG25-5A* model showed a sharp rise in RMSD, peaking around 17 Å. This suggests a highly flexible structure with significant conformational transitions occurring throughout the simulation.

The *StABCG25-5B* model displayed the highest RMSD values (~22 Å) among all, indicating substantial structural rearrangements and low overall stability. This model appears to have undergone major conformational changes, suggesting it is structurally less reliable. In contrast, the *StABCG25-6* model maintained an RMSD level around 3 Å throughout the simulation, emerging as the most stable model. Its low deviation indicates a high degree of structural integrity. Taken together, *StABCG25-2* and *StABCG25-6* stand out as the most stable structures based on their low RMSD values. In contrast, *StABCG25-5A* and especially *StABCG25-5B* showed high structural variability, suggesting they are unlikely to represent stable conformations under physiological conditions. These findings imply that such models may not reflect biologically relevant states. On the other hand, models like *StABCG25-3*, which began stabilizing during the later stages of the simulation, indicate that the current simulation time provided adequate sampling to capture their equilibrium conformations.

### 3.8. RMSF Analysis

Root-mean-square fluctuation (RMSF) analysis, one of the key outputs of molecular dynamics simulations, provides insight into the local flexibility of a system by quantifying how much each residue deviates from its average position over time. This analysis is particularly valuable for identifying highly mobile regions that may influence structural stability. RMSF results for all models are provided in Supplementary Materials, Figures S15–S21. The profiles for each model can be summarized as follows:

- The *StABCG25-1* model, which displayed substantial RMSD fluctuations throughout the simulation, showed RMSF values consistent with this structural flexibility. Notably, the N-terminal region (residues ~1–50) exhibited fluctuations exceeding 20 Å. Additional mobility was observed in mid-regions (~600–700) and near the C-terminus, suggesting widespread flexibility that aligns with the model’s overall structural instability.
- The *StABCG25-2* model, which showed minimal deviations in the RMSD analysis, also demonstrated limited fluctuations in the RMSF profile. Across the structure, most RMSF values remained within the 5–15 Å range, indicating strong local stability. Although minor flexibility was observed in certain loop regions (~200, ~800), these fluctuations were not significant enough to compromise the model’s overall structural integrity.
- The *StABCG25-3* model, which had reached a plateau in its RMSD curve after 400 ns, showed a concentrated fluctuation pattern in the RMSF analysis. Sharp peaks were observed in regions ~400–500, ~800–900, and ~1200–1300, indicating the presence of a few structurally flexible segments. The rest of the structure showed stabilized RMSF values around 3–5 Å, supporting the notion that the model had converged toward a stable conformation.
- The *StABCG25-4* model, which exhibited moderate RMSDs, revealed some localized flexibility in the RMSF analysis. Mobility was mainly seen in the N-terminal segment (~1–50) and around residue ~600. Overall, the model appears to be well-balanced, with restricted flexibility confined to a few regions.
- The *StABCG25-5A* model showed high structural mobility in both RMSD and RMSF analyses. Multiple regions exceeded 10 Å in RMSF, with the segments between ~100–200 and ~800–1000 displaying even more pronounced fluctuations.
- The *StABCG25-5B* model, which had the highest RMSD values among all, confirmed this dynamic behavior through the RMSF profile. Very high fluctuation values (20–25 Å) were found in the N-terminal region (residues 1–150), around residue ~800, and in some central segments (~400–500), reflecting extensive conformational rearrangements and a lack of structural integrity, similar to what was observed for *StABCG25-5A*.
- The *StABCG25-6* model emerged as the most stable in both RMSD and RMSF analyses. RMSF values remained within the narrow 3–7 Å range, and even the terminal regions did not exhibit significant mobility. This indicates a highly stable structure, both locally and globally, that preserved its conformational integrity throughout the simulation.

Taken together, the RMSF results align well with the RMSD findings. *StABCG25-2* and *StABCG25-6* demonstrated the highest levels of stability, both locally and globally. In contrast, *StABCG25-5A* and *StABCG25-5B* stood out as the most flexible models, suggesting limited stability under physiological conditions. Overall, the RMSF analysis offers important structural insight into how mobility in specific loops or terminal regions may influence the stability of the entire structure.

### 3.9. MMPBSA and MMGBSA Energy Calculations

MM/GBSA and MM/PBSA calculations performed for all models revealed that binding free energies are highly sensitive to solvent effects. The resulting ΔG scores are provided in Table 4, and detailed energy components are listed in the Supplementary Materials. (Sheet: GitHub Archive of Docking Inputs and Analysis Scripts: 4. all_models_mmpgbsa.dat). The observed differences in binding energies between the GB and PB methods are primarily attributed to the way solvent contributions are modeled. The GB approach estimates solvent effects using a simplified dielectric continuum, while the PB method computes electrostatic solvation more rigorously, capturing the desolvation penalty on binding in a more realistic manner. As reported in the literature [60,61], this can lead GB to overestimate binding affinity compared to PB. A striking example of this phenomenon was observed in the *StABCG25-5B* model. Here, the binding free energy predicted by GB was −44.52 kcal/mol, whereas PB yielded only −4.99 kcal/mol. In both methods, gas-phase contributions were nearly identical (ΔG_gas = −45.31 kcal/mol). However, solvent contributions differed substantially: −12.20 kcal/mol in GB vs. +40.67 kcal/mol in PB. Although *StABCG25-5a* and *5b* showed favorable binding energies in GB-based calculations, the RMSD and RMSF analyses indicated that these two models are unlikely to retain structural integrity under physiological conditions. In this context, our structural findings support the PB results over the GB ones.

## 4. Discussion

### 4.1. Originality of Annotation and Scope of the Study on ABCG25 Candidate Genes

Among candidates of the ABC transporter superfamily identified in our genomic scans, only *StABCG25-1*, *-2*, *-3*, and -4 showed strong and specific sequence similarity to *ABCG25.* This clear match strengthens both the originality and the focus of our study, suggesting that these isoforms in the potato (*Solanum tuberosum*) genome may retain the structural features of *Arabidopsis thaliana*’s well-characterized *AtABCG25* protein, especially its key role in abscisic Acid (ABA) transport. Based on this, the current study aims to explore in detail the role and regulation mechanisms of the *StABCG25* gene family in drought tolerance in potato.

### 4.2. Coordinated Activation of StABCG25 Genes and Drought Tolerance

The fact that *AtABCG25* single-gene mutants do not display a clear stomatal phenotype suggests that there may be functional redundancy among these transporters [5]. However, this redundancy may lead to different outcomes depending on the species or genotype, due to differences in the transcriptional regulation of these genes. In this study, the behavior of *StABCG25* candidate genes was analyzed across different tissues and genotypes (FB, Cardinal, and Toyoshiro) in potato.

### 4.3. Transcriptional Coordination and Redundancy Management

In the drought-tolerant FB clone, *StABCG25-2* and *StABCG25-4* isoforms showed consistent and coordinated activation under drought stress. This contrasts with the general suppression or inconsistent expression patterns observed in the sensitive genotypes. This regulation suggests that the functional redundancy among *ABCG25* transporters in potato is managed through transcriptional coordination. In particular, the simultaneous activation observed in the FB clone provides strong transcriptomic evidence for the individual functions of the *StABCG25* isoforms. It also points to their potential co-availability, which may enable heterodimerization or complex formation. These interactions are known to influence transporter efficiency, localization, and substrate specificity.

The function of the *ABCG25* transporter in ABA export depends on its ability to form either homodimers or heterodimers [62]. Recent structural and functional studies have clearly shown that *ABCG25* can heterodimerize with other ABCG transporters, such as *ABCG16*, to regulate its specific transport activity. For example, the heterodimerization of *ABCG16* and *ABCG25* has been reported to shift the transport substrate from ABA to its glucose-conjugated form, ABA-glucosyl ester (ABA-GE). This interaction also redirects the protein’s subcellular localization to the endoplasmic reticulum (ER) membrane [62].

### 4.4. Dominant Role of Transcriptional Regulation and Promoter-Controlled Expression

Although drought stress triggers widespread alternative splicing (AS) activity in the potato genome, no significant AS events were detected in the *StABCG25* genes. This may be related to the fact that *ABCG25* genes are involved in critical functions that require fast and immediate responses, such as stomatal closure. The lack of AS regulation for *ABCG25* proteins in the potato genome suggests a regulatory strategy that favors rapid adjustments in protein levels through transcriptional activation, rather than long-term structural changes through AS. This regulatory preference was further supported by the PlantCARE analysis, which was performed using 2000 bp promoter sequences of the *ABCG25* genes. In this analysis, several motifs identified in their promoter regions were found to be associated with jasmonate–ABA cross-regulation. In particular, the higher ABA scores calculated for *StABCG25-1* and *StABCG25-2* in this study point to well-known primary transcription factors involved in ABA signaling, such as bZIP (AREB/ABF, ABI5) [15,63], AP2/ERF (ABI4) [63], MYB/MYC [16] ve NAC (NAP) [8]. The fact that ABA concentration rises depending on the drought stress [16,64] triggers the TFs regulating ABA-sensitive genes [15]. These TFs stimulate the transcription of the *ABCG25* genes [1,8]. As a result, more ABCG25 genes are transcribed and embedded into the plasma membrane by forming homodimers [65,66]. This mechanism enables the export of ABA through *ABCG25* transporters, from the cells where it was synthesized, towards the apoplast [2,65]. Once in the apoplast, ABA is transported through the xylem to target tissues in the leaves, where it triggers stomatal closure and activates the drought response mechanism [5]. This finding supports the idea that *ABCG25* homologs are well-conserved through evolution and may function as stress sensors that respond rapidly to drought stress.

WGCNA analyses revealed that the roles of *StABCG25* paralogs differ within the gene co-expression network. *StABCG25-2* was positioned as a central hub gene in the turquoise module of the leaf tissue in the drought-tolerant FB clone, with a high module membership score (kME = 0.95). This centrality was not only associated with its expression level but also reflected by its strong functional connections within the network. In contrast, *StABCG25-4* and *StABCG25-3* showed negative correlations with their respective modules. This suggests that, depending on the biological context, these genes may act in opposition to overall module activation or may even play suppressive roles.

*StABCG25-4* was located in a module enriched with plastid-related GO terms (GO:0009536, GO:0042793), which may indicate that this gene is involved not only in classical ABA transport, but also in plastid-based metabolic or signaling pathways. Since plastids are key sites for ABA biosynthesis, the role of *StABCG25-4* may be more closely related to the transport of ABA precursors or intermediates within the plastid, rather than the export of ABA to the apoplast. This divergence strengthens the hypothesis that *StABCG25-2* (as a transcriptional hub) and *StABCG25-4* (as plastid-associated) may have evolved structural specializations to function in different subcellular compartments or to transport distinct substrates.

### 4.5. Genomic Coordination of Transcription Factors and Epigenetic Regulators

When examining regulatory partners, *StABCG25-2* in the FB clone showed strong co-expression with several stress-related transcription factors, including WRKY, AP2/ERF, and bZIP. The known ability of these TFs to bind ABA-responsive promoter motifs provides molecular-level support for the network-based findings. In contrast, in the tuber tissue of the Toyoshiro genotype, regulatory partners co-expressed with *StABCG25-4* and *StABCG25-1* included proteins associated with epigenetic regulation, such as SWI/SNF remodelers [67] and Jumonji domain-containing proteins [68]. This suggests that in sensitive genotypes, transcriptional repression may rely more on epigenetic mechanisms than on classic transcription factor–based regulation. In the literature, Jumonji-domain proteins are known to mediate gene silencing via histone demethylation [68], which could contribute to the suppression of *StABCG25* gene expression. These findings support the hypothesis that the key difference between tolerant and sensitive genotypes may not lie in cis-regulatory elements, but rather in the expression of trans-acting factors that control global chromatin accessibility and gene silencing.

### 4.6. Complexity in ABA Transport: Heterodimerization and Transporter Redundancy

*ABCG25* has been shown not to act alone, but rather to function together with other transporters such as *ABCG40*, *AIT1*, and *DTX50* to maintain ABA homeostasis [69,70]. More importantly, a recent study revealed that *ABCG25* forms a heterodimer with *ABCG16*, enabling selective substrate transport [62,64]. Such heterodimerizations can influence the tissue-specific localization of the transporter, its energy consumption, and the direction of transport (influx vs. efflux) [69]. Thus, the same gene may exhibit different functions in different tissues. The differences in expression and network dynamics among *StABCG25* paralogs may reflect this kind of molecular-level specialization. In light of current literature, the coordinated activation of *StABCG25-2* and *StABCG25-4* in the FB clone suggests the formation of a functional heterodimer (or a combined transporter complex) designed to operate in different compartments or with distinct substrates. Such cooperation may enable the tolerant genotype to establish ABA signaling more rapidly and efficiently. Upcoming molecular modeling and simulation analyses are expected to clarify the affinities and specificities of these *StABCG25* paralogs for ABA at the atomic level, thereby elucidating their heterodimerization potential and distinct functional roles (e.g., export vs. compartmental transport).

### 4.7. Structural Predictions and Docking Analyses

Structural modeling was performed using AlphaFold2-based predictions, which yielded reliable folding models for most isoforms (pLDDT > 75). These models were subjected to docking analyses to evaluate potential interactions with the ABA (abscisic acid) molecule. Notably, Model 5b exhibited low docking scores, indicating a strong binding potential to ABA. To quantitatively assess binding affinity, MM/GBSA and MM/PBSA free energy calculations were carried out, yielding divergent results. While MM/GBSA analysis suggested high-affinity binding, MM/PBSA predicted a weaker interaction. This discrepancy highlights the possibility of significant inconsistencies between methods, likely due to differences in how solvent polarity and electrostatic effects are modeled [61,71,72].

In our study, only the MM/PBSA results were consistently aligned with the structural stability data obtained from MD simulations. This further supports the notion that the PBSA method may offer a more reliable approach for modeling binding affinity, particularly under polar solvent conditions. In summary, molecular dynamics (MD) simulations uncover biologically critical details, such as time-dependent structural behavior and the sustainability of binding modes, that cannot be captured through static structures alone.

### 4.8. Structural Specialization and Divergence from Transcriptional Regulation

These structural evaluations revealed that the isoforms involved in ABA response differ not only at the transcriptional level, but also in their structural characteristics. The transport function of each *StABCG25* isoform appears to be specialized based on both its structural stability and expression profile.

#### 4.8.1. *StABCG25-6*: High Structural Stability, Low Transcription

The *StABCG25-6* isoform emerged as the most structurally stable model in molecular dynamics (MD) simulations, maintaining a consistent RMSD value of approximately 3 Å. However, RNA-Seq analyses in both leaf and tuber tissues revealed that this isoform is expressed at low levels. This suggests that *StABCG25-6* may perform a basal transport function rather than participating in rapid stress responses. It could be involved in maintaining ABA homeostasis or facilitating transport between small intracellular compartments, rather than in fast-response processes like stomatal closure, which require rapid ABA export. Its high structural integrity may reflect a function dependent on a fixed conformation, with a constitutive expression pattern maintained independently of external stimuli or time.

#### 4.8.2. *StABCG25-4*: High Expression, Dynamic Structure, and Plastid Association

In contrast, *StABCG25-4,* which is strongly activated in the drought-tolerant genotype, showed moderate flexibility in MD simulations, stabilizing around 7 Å RMSD. This level of flexibility is consistent with active transport cycles that require continuous conformational transitions between substrate binding and release. The protein’s dynamic behavior suggests an adaptation to fast transport cycles. Additionally, *StABCG25-4* was found within a co-expression module enriched for plastid-related transcriptional activity. Since plastids play a central role in ABA biosynthesis, this isoform may function not in exporting ABA to the apoplast, but in regulating the transport of precursors or ABA metabolites within the plastid. This points to a possible role in intracellular fine-tuning of ABA metabolism, rather than systemic ABA signaling.

### 4.9. Significance of an Integrated Approach and Functional Implications

These findings indicate that potato’s drought adaptation strategy relies not only on transcriptionally activated genes but also on structurally stable isoforms. For example, *StABCG25-2* and *StABCG25-6* stand out as key candidates in the ABA signaling pathway due to their structural robustness and expression patterns under drought conditions. This highlights the importance of integrating transcriptomic and structural data when characterizing gene function under stress. Therefore, this study not only identifies candidate genes associated with drought stress but also highlights the importance of integrating structure-based analyses with transcriptomic data to pinpoint functionally relevant isoforms. As emphasized in the literature, integrated approaches that combine high-resolution computational modeling with gene expression data have become indispensable tools for understanding the multilayered nature of biological function [73,74,75]. In conclusion, differences in structure and expression levels help us understand how each *StABCG25* isoform fulfills tissue-specific and specialized transport functions. This approach not only deepens our fundamental biological understanding but also offers new possibilities for targeted genetic improvement strategies.

## 5. Conclusions

This study employed a multilayered analytical framework to uncover the functional roles of the *StABCG25* gene family, which encodes ABA transporters in potato, within the context of drought adaptation mechanisms. The coordinated expression of *StABCG25-2* and *StABCG25-4* observed in the drought-tolerant genotype suggests that these genes may function not only individually, but also as part of a shared transport complex. Co-expression network analyses revealed that these genes occupy different centralities across distinct modules, with *StABCG25-2* emerging as a hub gene. Molecular dynamics simulations comparatively assessed the structural stability of these isoforms and provided a concrete link between expression level and structural fitness. In particular, *StABCG25-2* demonstrated both high expression and strong structural stability, reinforcing its candidacy as a robust ABA transporter. In contrast, the *StABCG25-6* isoform, although exhibiting low expression, maintained high structural integrity, indicating a likely role in basal-level transport functions. This integrative approach reinforces the importance of combining structural bioinformatics with transcriptomic data to identify targetable transporter genes.

### Future Perspectives

In future studies, the expression of these genes could be manipulated using genetic tools such as CRISPR/Cas9 or RNAi to directly assess their impact on drought tolerance. Furthermore, tissue-specific localization of *StABCG25* isoforms could be validated through immunohistochemistry or translational GUS/fluorescent protein tagging, providing critical insights for mapping transport routes. The heterodimerization potential of structurally stable isoforms should be evaluated using protein–protein interaction assays (e.g., Y2H, BiFC). Finally, the transport specificity of these isoforms for substrates other than ABA could be clarified through broader ligand screening studies.

## Figures and Tables

**Table 1:** List of *StABCG25*-like candidate genes with an ABA Score ≥ 8.

Gene ID	Transcript ID	^1^ Assigned Name	Total ABA Score ^2^
*PGSC0003DMG400001967*	*PGSC0003DMT400004973*	*StABCG25-1*	11
*PGSC0003DMG400015343*	*PGSC0003DMT400039671*	*StABCG25-2*	10
*PGSC0003DMG400019430*	*PGSC0003DMT400050007*	*StABCG25-3*	9
*PGSC0003DMG400000879*	*PGSC0003DMT400002293*	*StABCG25-4*	9
*PGSC0003DMG400000787*	*PGSC0003DMT400002061*	*StABCG25-5A*	9
*PGSC0003DMG400000787*	*PGSC0003DMT400002062*	*StABCG25-5B*	8
*PGSC0003DMG400019295*	*PGSC0003DMT400049667*	*StABCG25-6*	8

^1^ Since *PGSC0003DMT400002061* and *PGSC0003DMT400002062* are two isoforms of *PGSC0003DMG400000787*, their ABA scores were expressed as A and B for the models. ^2^ The entire scoring can be seen in Supplementary Materials, Table S6.

**Table 2:** Number of significant alternative splicing (AS) events detected under drought stress in different tissues and varieties (FDR < 0.05, |ΔPSI| > 0.1).

AS Event Type	Leaf (Cardinal)	Leaf (FB Clone)	Tuber
SE (Skipped Exon)	10	57	80
RI (Retained Intron)	46	212	170
MXE (Mutually Exclusive Exons)	0	4	7
A5SS (Alt. 5′ Splice Site)	13	34	29
A3SS (Alt. 3′ Splice Site)	42	72	59
Total	111	379	345

**Table 3:** AlphaFold2 Quality Metrics for Predicted Models.

Model	Coverage	PAE Intra	PAE Inter	pLDDT Avg.	pLDDT Consistency	Confidence
*StABCG25-1*	High	Yes	Yes	85	High	Good overall
*StABCG25-2*	High	Yes	Yes	80	High	Good overall
*StABCG25-3*	High	Yes	Yes	78	High	Good overall
*StABCG25-4*	High	Yes	Partial	82	High	Good overall
*StABCG25-5a*	High	Yes	No	76	High	Good overall
*StABCG25-5b*	High	Yes	Partial	79	High	Good overall
*StABCG25-6*	High	Yes	No	77	High	Good overall

**Table 4:** Energy calculation results for the models (kcal/mol).

Models	Docking	MMPBSA	MMGBSA
Model 1	−5.9	−4.6483	−15.9602
Model 2	−6.3	−4.2699	−26.0802
Model 3	−6.4	2.0634	−16.3797
Model 4	−6.2	2.2088	−24.0798
Model 5a	−6.3	−2.651	−36.6501
Model 5b	−6.6	−4.9877	−44.5229
Model 6	−6.2	−6.3511	−37.1078

## Data Availability

All data supporting the findings of this study, including raw expression matrices, gene co-expression module assignments, protein–ligand docking outputs, and GO/KEGG enrichment results, are provided as Supplementary Materials. In addition, all custom scripts, input data, and analysis pipelines used in this study are publicly available at the following GitHub repository: https://github.com/bkurt00/ABCG25_md (accessed on 16 July 2025).

## Supplementary Materials

The following supporting information can be downloaded at: https://www.mdpi.com/article/10.3390/biology14121723/s1, Figures S1–S7: AlphaFold2 model confidence metrics (pLDDT, PAE, and sequence coverage) for *StABCG25* isoforms; Figures S8–S14: Docking poses of *StABCG25* models showing overall binding pocket, 3D interaction visualization, and 2D ligand–protein contact maps; Figures S15–S21: Residue-based RMSF profiles of AlphaFold2-predicted *StABCG25* models; Tables S1 and S2: BLASTP search results of AtABCG25 against the *Solanum tuberosum* proteome; Table S3: Correspondence of protein and transcript IDs for *StABCG25* candidates; Table S4: Conserved domain and motif search results; Tables S5 and S6: PlantCARE promoter motif annotations and ABA scoring results; Table S7: Molecular dynamics simulation workflow and parameters (start_md.py); Tables S8–S10: Co-expressed genes interacting with *StABCG25* family members in the leaf tissue of the drought-tolerant FB clone; Table S11: Gene Ontology (GO) enrichment results for WGCNA modules containing *StABCG25* genes in leaf tissue; Tables S12 and S13: Co-expressed genes interacting with *StABCG25* family members in the tuber tissue of the drought-sensitive Toyoshiro cultivar; Table S14: GO enrichment results for WGCNA modules containing *StABCG25* genes in tuber tissue; Sheet: GitHub Archive of Docking Inputs and Analysis Scripts—includes prepare_box.py, start_md.py, and MM/PBSA configuration files.

## Abbreviations

A3SS, Alternative 3′ Splice Site; A5SS, Alternative 5′ Splice Site; AAC, Amino Acid Catabolism; ABA, Abscisic Acid; ABC, ATP-binding cassette; AS, Alternative Splicing; AtABCG25, *Arabidopsis thaliana* ABCG25 Transporter; DEAACs, Differentially Expressed Amino Acid Catabolism Genes; ΔΨ, Percent Spliced In difference; FDR, False Discovery Rate; GO, Gene Ontology; kDiff, Centrality Difference; kME, Module Membership; kOut, Out-Module Connectivity; kTotal, Total Connectivity; kWithin, In-Module Connectivity; LRR, Leucine-rich repeat; MD, Molecular Dynamics; MM/GBSA, Molecular Mechanics/Generalized Born Surface Area; MM/PBSA, Molecular Mechanics/Poisson–Boltzmann Surface Area; MSA, Multiple Sequence Alignments; MXE, Mutually Exclusive Exons; OST1, Open Stomata 1; PAE, Predicted Aligned Error; pLDDT, Predicted Local Distance Difference Test; PKs, Protein Kinases; RI, Retained Intron; RLKs, Receptor-like Kinases; RMSD, Root-Mean-Square Deviation; RMSF, Root-Mean-Square Fluctuation; SE, Skipped Exon; *StABCG25*, *Solanum tuberosum* ABCG25 Transporters; StASN, Asparaginase; StCPK9, *Solanum tuberosum* Calcium-dependent protein kinase 9; StHMGL, Hydroxymethylglutaryl-CoA lyase; TCA, Tricarboxylic Acid Cycle; TFs, Transcription Factors; TOR, Target of Rapamycin; WGCNA, Weighted Gene Co-expression Network Analysis.
